# Ground-Level Fall vs. Other Mechanisms of Injury in Patients With a Shock Index of Greater Than One at a Rural Level 1 Trauma Center

**DOI:** 10.7759/cureus.108716

**Published:** 2026-05-12

**Authors:** Jacob S Snipp, Samuel D Cartwright, Maria Eckmann, Matthew Leonard, Hannah Wheeler, Bracken Burns, Kevin H Zeng, Eric Olsen, Alexander Mays

**Affiliations:** 1 Trauma Surgery, East Tennessee State University Quillen College of Medicine, Johnson City, USA; 2 Trauma, Ballad Health, Johnson City, USA

**Keywords:** geriatric trauma, ground level fall, injury severity score, mechanism of injury, rural trauma center, shock index, triage protocol

## Abstract

Ground-level falls (GLFs) often are viewed as minor injuries but can pose significant risks for patients with a shock index (SI) greater than one. This retrospective study is aimed at comparing outcomes of GLF patients with a high SI to those who sustained trauma via other mechanisms who also have a SI>1 to provide insight for improved trauma care. Conducted at Johnson City Medical Center in Johnson City, Tennessee, from September 1, 2021, to January 1, 2024, the study analyzed data from a trauma registry and EpicCare Link (Epic Systems Corporation, Verona, USA), focusing on demographics, injury details, and clinical outcomes. 387 patients met the inclusion criteria. 16.5% (n=64) of patients comprised the GLF population, were older (mean of 65.6 vs 43.6 years, p<0.001), predominantly female (p<0.001), and more likely referred from other facilities (p<0.001). They had lower injury severity scores (ISS) (p=0.002), lower emergency medical service (EMS) shock index scores (p=0.012), and were less likely to need surgery (p=0.007). All patients had an SI>1. Despite these differences, mortality rates were similar to those of other trauma patients, emphasizing the critical nature of GLFs despite their seemingly benign mechanism.

## Introduction

Ground-level falls (GLFs) represent a significant public health concern, contributing substantially to injury-related deaths, particularly among older adults. GLFs are defined as a fall resulting in contact with the ground at the same level at which the fall originated, excluding falls from any height. A recent review of the National Trauma Data Bank supports GLFs trend with age [1]. This is critical as the United Nations projects that by the mid-2030s, there will be 265 million individuals aged 80 and older, which will surpass the projected population of infants globally [2]. In the United States, falls account for over 34,000 fatalities annually in individuals aged 65 and older [3]. Adults who sustain GLFs often have underlying comorbidities like cardiovascular disease, dehydration, and polypharmacy, which may complicate severity understanding through independent effects on hemodynamic stability. These factors may mask severity in the setting of seemingly low-energy mechanisms, particularly when evaluating hemodynamic markers such as shock index (SI). GLFs, while often perceived as a less severe mechanism of injury, can still lead to serious consequences, especially in patients presenting with an SI>1, and often require trauma activation. Trauma activation represents a serious level of trauma response and is reserved for patients who present in critical physiologic instability, which requires immediate multidisciplinary action. The SI is calculated by dividing heart rate by systolic blood pressure and serves as a valuable clinical tool for assessing hemodynamic status and predicting mortality in trauma patients. An SI greater than one indicates a high-risk state, even when the apparent mechanism of injury might suggest otherwise [4].

While many current studies evaluate screening tools such as cervical spine assessment, the first thing assessed in every fall [5], and risk stratification of anticoagulant use [6], there is a relative paucity of data specifically comparing outcomes for GLF patients with an SI>1 to those sustaining injuries from other mechanisms, despite the growing burden of GLFs on an aging population. SI could serve as a rapid bedside tool that helps to identify GLF patients at higher risk of mortality despite the perceived low-energy mechanism. This discrepancy underscores the need for further investigation into the specific risks associated with GLFs in the context of an SI>1. This study sought to examine the outcomes of patients presenting with an SI greater than one following a GLF compared to those with an SI>1 from other traumatic mechanisms of injury. Outcomes included: necessity of surgical intervention, intensive care unit (ICU) length of stay, hospital length of stay, ventilator days, blood product usage, and discharge disposition, which are fundamental to understanding trauma mechanism severity. This objective was chosen with the presumption that GLF patients with an SI>1 would demonstrate comparable mortality and clinical outcomes to patients sustaining perceived higher-energy mechanisms of trauma with a similar SI, further emphasizing SI as a reliable indicator of clinical instability despite perceived trauma mechanism. 

This IRB-approved, retrospective study was previously presented at the 2025 South Eastern Surgical Conference (SESC) on February 18th, 2025. This study analyzed adult patients admitted as trauma activations between September 1, 2021, and January 1, 2024, at Johnson City Medical Center, a Level I Trauma Center in a rural area. Trauma activations identify patients with significant potential for severe injury requiring immediate evaluation and intervention and represent the highest levels of trauma. 

## Materials and methods

Data were collected by researchers associated with East Tennessee State University retrospectively from the Johnson City Medical Center Ballad Health trauma registry and the electronic health record system - EpicCare Link (Epic Systems Corporation, Verona, USA). The inclusion criteria were: (1) age ≥18 years, (2) admission as a trauma activation, and (3) a documented SI greater than one upon initial evaluation in the trauma bay. Patients were excluded if they were seen only as trauma consults, transported by non-emergency medical services (i.e., private vehicle), had a documented SI<1, or lacked complete data regarding SI or mechanism of injury. 

Patients were categorized into two groups based on their mechanism of injury: GLF and other trauma mechanisms (OTM). GLF was defined as a fall resulting in contact with the ground at the same level from which the fall originated (i.e., not from a height). OTM included all other mechanisms of injury, such as assault, animal-related injury, burn injuries, machinery injuries, motorcycle collisions, motor vehicle collisions, knife wounds, falls from height, gun injuries, blunt force trauma, bicycle accidents, all-terrain vehicle (ATV) accidents, and pedestrian accidents. Specific etiology of GLF, such as syncope, mechanical fall, or neurovascular event, was not obtained as a discrete variable in this study and is acknowledged as a possible limitation. Data elements collected were demographics (age, gender), clinical parameters [emergency medical service (EMS) shock index, injury severity score (ISS)], hospital course variables (ICU length of stay, hospital length of stay, ventilator days, units of blood transfused, requirement for surgical intervention), logistical variables (patient origin, mode of transportation), and patient outcomes (hospital mortality, discharge disposition). Surgical interventions were captured as a binary variable of either "yes" or "no" during the index admission and were not stratified by type, anatomical region, or direct relationship to the traumatic mechanism of injury.

Patient comorbidities, medication use, and prior medical history were not systematically collected as discrete variables in this study. Given the older demographic of the GLF cohort, future investigations could incorporate validated frailty indices, comorbidity scores such as the Charlson Comorbidity Index, and medication profiles, particularly anticoagulant and antihypertensive use, for better characterization of the physiologic vulnerability of this population and its influence on SI and clinical outcomes. Statistical analysis was performed using independent-samples t-tests to compare continuous variables between the two groups (GLF and OTM). Categorical variables were compared using Chi-square tests. A p-value of less than 0.05 was considered statistically significant. Data were analyzed using JASP V0.19 (University of Amsterdam, Amsterdam, The Netherlands). 

## Results

A total of 387 patients met the inclusion criteria. Of these, 16.5% (n=64) sustained a GLF. GLF patients were significantly older (65.6±16.5 years vs. 43.6±18.0 years, p<0.001) and more likely to be female compared to other mechanisms [56.3% (n=36) vs. 31.9% (n=103), p<0.001]. GLF patients were also more frequently transferred from an outside facility [37.5% (n=24) vs. 13.1% (n=42), p<0.001] and had a significantly longer ICU length of stay (p=0.015). While GLF patients presented with a lower EMS shock index (1.2±0.18 vs. 1.3±0.30, p=0.012) and had lower ISS (10.8±10.3 vs. 16.7±13.2, p=0.002), they were less likely to require surgical intervention during their admission [30% (n=19) vs. 48% (n=155), p=0.007]. EMS shock index is calculated using the initial vitals obtained en route to the hospital and gives insight into the patient's status during transport. No significant differences were observed between the two groups with respect to length of hospital stay (p=0.243), ventilator days (p=0.058), units of blood products transfused (p=0.051), or in-hospital mortality (p=0.974). On discharge, GLF patients were more likely to be discharged to a care facility compared to patients with other injury mechanisms [36.5% (n=23) vs. 18.1% (n=58), p<0.001] (Table 1). 

**Table 1:** GLFs Compared to Other Injury Mechanisms Shock Indexes ≥1 Continuous variables collected using independent-samples t-tests are described as mean±standard deviation, and categorical variables collected using Chi-square tests are reported as frequency (%). Continuous variables with no chi-square values and categorical variables with no t-test values are denoted with (-). Data were analyzed using JASP V0.19. GLF: ground-level falls; LOS: length of stay; ICU: intensive care unit; ISS: injury severity scores; EMS: emergency medical service.

Variable	Other (N=323)	GLF (N=64)	p-value	t-test value	Chi-squared value
Age, years	43.60±18.04	65.64±16.49	<0.001	-9.052	(-)
Hospital LOS, days	10.1±13.2	8.0±8.8	0.243	1.219	(-)
ICU LOS, days	7.5±7.3	5.2±4.1	0.015	2.444	(-)
Ventilator Days	5.3±6.1	3.8±3.6	0.058	1.901	(-)
ISS	16.7±13.2	10.8±10.3	0.002	3.377	(-)
EMS Shock Index	1.3±0.3	1.2±0.2	0.012	2.555	(-)
Gender, male	220 (68.1%)	28 (43.7%)	<0.001	(-)	12.735
Patient Origin			<0.001	(-)	20.962
Scene	281 (86.9%)	40 (62.5%)		(-)	
Referring Facility	42 (13.1%)	24 (37.5%)		(-)	
Mode of Transportation			0.007	(-)	6.327
Ground Ambulance	257 (79.5%)	60 (93.8%)			
Helicopter	66 (20.5%)	4 (6.2%)			
Surgical Intervention	155 (48.0%)	19 (30.0%)	0.007	(-)	15.709
Units of Blood Products Transfused	97 (30%)	6 (9.1%)	0.051	(-)	10.635
Mortality	51 (15.8%)	10 (15.6%)	0.974	(-)	0
Discharge to Care Facility	58 (18.1%)	23 (36.5%)	<0.001	(-)	9.377

## Discussion

The findings of this study challenge the perception of GLFs as a less severe injury mechanism in patients with an SI>1, highlighting the importance of considering SI regardless of the mechanism of injury. Despite GLFs appearing to be a less severe mechanism of injury, patients who present with a GLF and SI >1 have similar outcomes compared to OTM. Mortality rates were found to be nearly identical between the two groups, which is a clinically profound finding. A GLF with an SI>1 is a known strong predictor of mortality among the elderly population, which could be responsible for the mortality similarity found in this study [7]. This is a previously identified phenomenon known as the geriatric trauma paradox, in which older patients have higher mortality rates than younger patients despite a similar ISS and mechanism of injury (MOI) [8-10]. Older age acts as a physiological multiplier with mortality risk due to decreased physiologic reserves and a higher number of comorbidities, despite mechanisms of injury that may appear less severe. Prior research has demonstrated that elderly patients who sustain GLFs face a mortality risk similar to higher energy trauma mechanisms such as motor vehicle crashes despite the significant disparity in energy between the two mechanisms [11]. No significant differences in mortality between GLF with an SI>1 and other mechanisms in this study could also be the result of GLF patients presenting with perceived lower severity injuries being assigned less triage priority, delaying time to definitive care. 

GLF patients were also more likely to be discharged to a care facility after hospitalization, which highlights GLF mechanisms as more severe over time than what is normally perceived, which implies patients' long-term functional recovery may be worse. In support of worsened long-term functional recovery, one study found that patients discharged to skilled care facilities post GLF mechanism were at threefold higher risk for one-year mortality [12, 13]. These results advocate for a more nuanced approach to trauma triage and management. Current trauma protocols often deprioritize GLF patients based solely on the presumed low-energy nature of the mechanism. The data from this study suggest that GLF patients with an SI>1 warrant equal attention and resource allocation, including prompt critical care assessments and aggressive monitoring to not only save lives but to preserve quality of life after injury. 

Furthermore, the absence of increased surgical interventions or transfusion requirements in GLF patients supports the hypothesis that their hemodynamic instability stems from factors other than acute hemorrhage. This finding points towards SI being more of a marker of frailty than bleeding in geriatric populations. Unlike in younger trauma patients with an SI >1 who are almost exclusively associated with overt exsanguination, previous studies suggest that in elderly patients, their physiological state may be driven more by things such as occult sepsis, chronic dehydration, or cardiovascular changes [7, 8, 14]. Shock index has been demonstrated as a highly reliable correlate for outcomes across most age groups, maintaining predictive integrity until the octogenarian stage, at which point age-related physiological changes may further complicate clinical assessment [15, 16]. Given that the mean age in the GLF cohort was 65.6 years, the findings suggest that an SI>1 remains a potent indicator of clinical instability and should not be discounted due to the perceived low-energy nature of the mechanism. 

Several limitations should be acknowledged. This study was conducted at a single center, limiting the generalizability of the findings to other settings. Comparisons of patients presenting with an SI<1 were not conducted. SI>1 was chosen primarily to eliminate possible triage bias in which patients might be stratified lower due to the mechanism of trauma, delaying care in areas like ICU stays and mortality. The retrospective design is also prone to selection bias and may limit the ability to control unmeasured confounders. The etiology of individual ground-level falls was not recorded, which may represent a confounding variable, given that the underlying cause of a fall, whether mechanical, syncopal, or medication-induced, may independently influence hemodynamic status and clinical outcomes. Furthermore, surgical interventions were captured as a binary variable and were not stratified by type or direct relationship to the traumatic mechanism. This limits the ability to conclude the specific operative needs of GLF patients with an SI >1 compared to those sustaining other traumatic mechanisms, as some surgical procedures may have been performed for indications unrelated to the index trauma. Another limitation was the absence of patient comorbidity and medication data. In an elderly GLF population, underlying conditions such as cardiovascular disease, anticoagulation use, and frailty may independently contribute to hemodynamic instability and elevated SI values, potentially confounding comparisons between GLF and OTM cohorts. The absence of a validated frailty index or comorbidity score represents an important gap that future prospective studies should address. Institutional variability in trauma activation criteria across different centers was an acknowledged limitation in generalizability and reproducibility in this study. Trauma activation thresholds may vary greatly between institutions, EMS agencies, and geographic regions. The inclusion criteria of this study required trauma activation status, which is tied to specific activation protocols at Johnson City Medical Center and surrounding EMS agencies. 

## Conclusions

Prospective studies are essential to validate these findings across diverse patient populations and settings. Further investigation into the pathophysiological mechanisms driving SI and GLF patients is also warranted. Specifically, comparing SI from EMS vs SI on hospital admission would also be useful in better learning the differences in patient care dynamics. Understanding the contributions of comorbid conditions, age-related cardiovascular changes, and medication effects will be crucial in developing tailored management strategies. Additionally, future investigations comparing GLF patients with an SI<1 to those with an SI>1 may further illustrate the severity of GLF regardless of a patient's pre-disposed SI. Moreover, research into the role of SI-guided interventions in the population has the potential to improve outcomes. SI>1 in GLF patients may not indicate hemorrhagic shock, as evidenced by their lower need for surgery and transfusion. However, these patients are still critically ill, as indicated by similar mortality outcomes compared to other trauma patients. These findings challenge the notion that GLFs are inherently less dangerous, emphasizing the importance of assessing SI in all trauma patients, regardless of injury mechanism. 
